# Level of Expression of the Nonmutant *Ferrochelatase* Allele is a Determinant of Biochemical Phenotype in a Mouse Model of Erythropoietic Protoporphyria

**DOI:** 10.4137/grsb.s636

**Published:** 2008-05-29

**Authors:** Joseph Bloomer, Yongming Wang, Dongquan Chen

**Affiliations:** 1 From Division of Gastroenterology/Hepatology and; 2 Division of Hematology/Oncology Department of Medicine, University of Alabama at Birmingham School of Medicine, Birmingham, Alabama

**Keywords:** erythropoietic, protoporphyria, mouse, genotype/phenotype

## Abstract

Ferrochelatase (FECH) activity is decreased in erythropoietic protoporphyria (EPP), causing increased production and excretion of protoporphyrin. This study examined whether the level of expression of the nonmutant *FECH* allele is a determinant of phenotype in a mouse model of EPP that carries a heterozygous deletion of exon 10 in *FECH*. Two mice strains that had a two-fold difference in *FECH* mRNA levels in bone marrow and liver (low expressing C3H/HeJ and high expressing CBA/J) were used to establish congenic strains containing the mutation. Erythrocyte protoporphyrin levels in C3H/HeJ heterozygous mice were significantly higher than in their wildtype littermates, whereas levels in CBA/J heterozygous mice did not differ significantly from their wildtype littermates. Biliary excretion of protoporphyrin was also significantly higher in C3H/HeJ heterozygous mice. The levels of normal *FECH* mRNA in bone marrow measured by real time PCR were 138 +/− 30 copies per ug total RNA in C3H/HeJ +/− mice, 320 +/− 59 in C3H/HeJ +/+ mice and 634 +/− 38 in CBA/J +/+ mice. Levels in liver tissue of the mice differed significantly in the same pattern. Thus, the level of expression of the nonmutant *FECH* allele is a determinant of phenotype in a mouse model of EPP as has been demonstrated in human EPP.

## Introduction

Erythropoietic protoporphyria (EPP) is a genetic disorder of porphyrin metabolism in humans that was first described in 1961 by Magness and co-workers when they reported a man with lifelong sensitivity to sunlight and increased protoporphyrin levels in erythrocytes and feces (Magness et al. 1961). Photosensitivity, which is the major clinical feature, results from the photoactive damage of protoporphyrin to skin (Timonen et al. 2000). Some patients also develop hepatobiliary disease due to protoporphyrin induced damage to liver structure and function (Doss and Frank, 1989). This may progress to liver failure and necessitate liver transplantation (McGuire et al. 2005).

A deficiency of ferrochelatase (FECH, EC4.99.1.1) activity underlies the excessive production and excretion of protoporphyrin in EPP (Bonkovsky et al. 1975). FECH, which is the last enzyme in the heme biosynthesis pathway, is located on the matrix side of the inner mitochondrial membrane and catalyzes the insertion of ferrous iron into protoporphyrin to form heme (Dailey et al. 2000). All heme forming tissues may potentially contribute to the excess formation of protoporphyrin in EPP, with the bone marrow being the major source (Poh-Fitzpatrick, 1985). The functional human FECH enzyme is a homodimer that contains two NO sensitive and coordinated 2Fe-2S clusters (Wu et al. 2001). The *FECH* gene contains 11 exons, and *FECH* mRNA has an open reading frame of 1269 bases that encodes a protein of 423 amino acid residues (Nakahashi et al. 1990). There is a single transcript with 2 poly-adenylation sites in erythroid and non-erythroid cells, indicating there is only one *FECH* gene in heme forming tissues (Taketani et al. 1992).

Patients with EPP are usually heterozygous for a *FECH* mutation, even those with severe disease, and extensive genetic heterogeneity has been noted (Wang et al. 1994; Rufenacht et al. 1998). The gene mutations by themselves do not account for disease expression, as they cause only minimally increased protoporphyrin overproduction (Chen et al. 2002). Thus most individuals with symptomatic disease also have a polymorphism in intron 3 of the nonmutant *FECH* allele (IVS3–48c) that lowers gene expression (Gouya et al. 2002; Risheg et al. 2003).

The purpose of this study was to examine whether the level of expression of the nonmutant *FECH* allele is also a determinant of phenotype in a mouse model of EPP, using mice in which an exon 10 deletion of *FECH* had been introduced into the genome of mouse embryonic stem cells by homologous recombination (Magness and Brenner, 1999; Magness et al. 2002).

## Methods

### Selection and breeding of Mice

Magness and Brenner provided mice carrying the exon 10 deletion, along with a neomycin resistant cassette in the same allele (Magness et al. 2002). Primers were designed to hybridize with this cassette, and PCR amplification of genomic DNA from animals with the exon 10 deletion produced a product that was identified by ethidium bromide stain on agarose gel, whereas genomic DNA from wild-type animals did not produce the product. Wild-type strains were selected on the basis of *FECH* gene expression as assessed by the level of *FECH* mRNA in liver and bone marrow, using relative quantitative RT-PCR as previously described (Risheg et al. 2003). Mice from five different strains were evaluated (Table 1). Based on the results, CBA/J mice were selected as a high expressing strain and C3H/HeJ mice as a low expressing strain. *FECH* cDNA and 1000 bases of the *FECH* gene promoter and 5′ untranslated region of the two mice strains were sequenced, and no differences were found. Intron 3 was also sequenced, and both strains had base t at position -48. Thus it is likely that the difference in *FECH* expression in the two strains is caused by transacting factors and/or modifier genes, not by a difference in *FECH* gene sequence.

In order to produce congenic strains, male offspring carrying the exon 10 deletion were back-crossed with wild-type female C3H/HeJ and CBA/J mice through 7–9 generations. Breeding of the mice was carried out in the University of Alabama at Birmingham Genetic Engineered Mouse Breeding Facility. The biochemical phenotype of heterozygous adult mice (3–6 months old) and their wild-type littermates was assessed by measurement of 1) erythrocyte protoporphyrin level; 2) total porphyrin level and fluorescence peak in bile; 3) FECH enzyme activity in liver. The normal *FECH* mRNA level in bone marrow and liver was measured by quantitative real time PCR. Statistical analysis was done by the students T test and Wilcoxin/Kruskal-Wallis test.

### Biochemical measurements

Protoporphyrin levels in erythrocytes were measured by spectroflourometry after solvent partitioning (Morton et al. 1988; Bloomer et al. 1998). The level and fluorescence peak of porphyrin in bile was assessed by spectroflourometry after dissolving the bile in perchloric acid methanol (Morton et al. 1988; Bloomer et al. 1998). Bile was obtained from gallbladders of the mice at the time of sacrifice.

The level of FECH activity in whole liver homogenates was measured by Zn-deuteroporphyrin formation in pmol per second per mg protein (Bloomer et al. 1998).

### Quantitative measurement of *FECH* mRNA by real-time PCR

The level of *FECH* mRNA was measured in total RNA extracted from whole liver tissue and from bone marrow of leg bones of the adult mice. Only the level of the wild-type (normal) species of *FECH* mRNA was measured in wild-type mice and mice heterozygous for the exon 10 deletion in *FECH* gene. The mutant species of *FECH* mRNA was not measured. The method used the Quantitect™ custom assay developed by Qiagen (Valencia, CA).

In order to develop the standard curve for the assay, RNA was extracted from mouse liver, and a 484 bp *FECH* amplicon (from exon 7 to exon 11) was generated by RT-PCR. The primer sequences were sense, TCATCCAGTGCTTTGCAGAC; antisense, AGCTTGTTGGACTGGATGTG. The amplicon was purified on silica column (QIA quick PCR purification, Qiagen) and cloned into pCRS.1 vector (Invitrogen). Ligated fragments were placed in DH5a competent cells (Life Technologies). Plasmid DNA was prepared and the cloned amplicons were sequenced; cDNA plasmid concentrations were measured by optical density spectrophotometry (Spectronic Genesys 5). Copy number determination was calculated using the following formula: Copies/ml = 6.023 × 10^23^ × [C] × OD_260_/molecular weight Where [C] = 5 × 10^−5^ g/ml for DNA; Molecular weight of the PCR product = number base pairs × 6.58 × 10^2^. Serial dilutions from the cDNA plasmid were used as standard curves.

Primers and probes were designed by Qiagen Quantiprobe Design Software (Qiagen). The probe was designed to locate in exon 10 for specific detection of wild-type *FECH* mRNA. The probe sequence was ATCAGAAGAGCGGAGT with fluorophone at the 3′ end and nonfluorescent quencher and minor grove binder (MGB) at the 5′ end. The PCR primers yield a 93-nt amplicon from wild-type *FECH* with one primer specific to exon 9/10 fusion region and one primer specific to exon 11. Real-time PCR was performed on an ABI prism 7700 sequence detection system (Applied Biosystems). PCR conditions were 95 °C for 15 min, 40 cycles at 94° for 15s, 56 °C for 30s and 76 °C for 30s. Each 50 μl reaction contained 25 μl 2× QuantiTect probe PCR Master Mix, 2.5 μl 20× primer mix, 2.5 μl 20 × Quantiprobe Solution and template cDNA. Each sample was run in triplicate. Standard wells contained 4000, 2000, 1000, 500, 200, 100, 50 and 10 copies of plasmid *FECH* cDNA.

### DNA microarray analysis of bone marrow gene expression

Microarray analysis of bone marrow gene expression in C3H/HeJ mice and CBA/J mice was done using the GeneChip® Mouse Expression Set 430 (Affymetrix, Santa Clara, CA). Bone marrow was obtained from leg bones of 3 adult male mice and pooled as a single sample. Three samples were prepared for the two mice strains, total RNA was isolated by TRIzol reagent (Life Technologies, Rockville, MD) from each of the 6 samples, and 2 ug of total RNA from each sample was submitted to the Microarray Shared Facility for Affymetrix Gene Chip Analysis, University of Alabama at Birmingham Comprehensive Cancer Center.

The details of the procedures used in the analysis are presented in the manufacturer’s technical manual (Affymetrix). In brief, the quality of the RNA was determined using the RNA Nano Chip on the Agilent BioAnalyzer before synthesis of double-strand cDNA. Double-strand cDNA was generated by linear amplification using an oligo dT-T7 primer and reverse transcriptase (RT). Biotin labeled cRNA was then synthesized by in vitro transcription (IVT) using the 3′-Amplification Reagents for IVT labeling (Affymetrix). The quality of the cRNA was determined on the Agilent BioAnalyzer before it was fragmented into 50 to 200 base fragments. Prior to hybridization to the expression arrays, the quality of the hybridization target was determined by hybridization to a Test3 array. The result indicated the efficacy of the RT/IVT reaction by the ratios of expression level of 5′ to 3′ of the housekeeping genes β-actin and GAPDH. If the quality of the hybridization target passed the quality parameters from the Test3 array, the expression arrays were hybridized overnight at 45 °C and then washed, stained, and scanned on the following day. Gene expression levels were extracted using the Gene Chip Operating Software. Comparisons were made of bone marrow gene expression for each of the 3 CBA/J samples with each of the 3 C3H/HeJ samples to determine the fold change for each transcript (9 total comparisons).

For microarray data analysis and annotation, the software packages GeneTraffic (Iobio/Stratage/Agilent, Inc. Santa Clara, CA) and ArrayAssist Enterprise together with PathwayAssist (Stratagene/Agilent, Santa Clara, CA) were used. Briefly, the raw Genechip files from GCOS were uploaded, background-subtracted, and normalized with GC-RMA method (Gentleman et al. 2004). The control group was used as a baseline to calculate the intensity ratio/fold changes of the treated group versus the control group. The ratio was log2-transformed before further statistical analysis. The p-values were obtained by an unpaired t-test assuming unequal variance.

## Results

Levels of erythrocyte protoporphyrin in adult (3–6 months old) heterozygous C3H/HeJ mice were significantly higher than in C3H/HeJ wild-type mice, CBA/J heterozygous mice and CBA/J wildtype mice (Table 2). There was no significant difference noted between male and female mice in the same strain. Bile porphyrin levels in the heterozygous C3H/HeJ mice were also significantly increased compared to C3H/HeJ wild-type mice (79 ± 0.1 versus 28 ± 0.1 ug/dl, p = 0.006), and the peak emission fluorescence was at 604 nm, characteristic of protoporphyrin. Thus, there was increased production and excretion of protoporphyrin in the heterozygous C3H/HeJ mice.

The levels of normal *FECH* mRNA in bone marrow and liver of wildtype C3H/HeJ mice were approximately 50% of those in CBA/J wildtype mice (Table 3), which agreed well with the relative levels estimated by relative quantitative PCR (Table 1). The lowest levels of normal *FECH* mRNA were found in C3H/HeJ heterozygous mice, in which bone marrow and liver levels were reduced by 57% and 60% respectively compared to levels in wildtype C3H/HeJ mice.

FECH enzyme activity in livers of heterozygous C3H/HeJ mice was reduced by 53% compared to the activity in wildtype C3H/HeJ mice, in parallel with the reduction in normal liver *FECH* mRNA. This argues against a significant dominant/negative effect of the mutant FECH protein on the normal protein in mice with an exon 10 deletion. If that were the case, the reduction in FECH enzyme activity should have been approximately 75%.

In order to compare bone marrow expression of other genes in these two mouse strains, and potentially to identify candidate modifier genes of *FECH*, DNA microarray analysis was done in adult male CBA/J and C3H/HeJ mice. This identified 103 genes that were upregulated in CBA/J mice compared to C3H/HeJ mice as defined by at least a 1.5 fold increase in mRNA level (p < 0.05) (Table 4). There were 31 genes which had more than a two-fold change in the level of expression. In contrast there were only 48 bone marrow genes that were upregulated in C3H/HeJ mice compared to the CBA/J mice, six of which had a more than two-fold change in level of expression (Table 5). Thus more bone marrow genes in CBA/J mice are upregulated than in the C3H/HeJ mice. The pathways that were most impacted by these changes were SAPK-JNK signaling, mitrochondrial apoptosis control, integrin signaling, death receptor signaling, caspase signaling, apoptosis, PDGF signaling. The genes encoding other enzymes of the heme biosynthesis pathway showed no significant difference in level of expression between C3H/HeJ mice and CBA/J mice.

## Discussion

This study demonstrates that mice strains have different levels of *FECH* mRNA in the major tissues of heme formation, bone marrow and liver, as CBA/J mice had levels approximately two times those found in C3H/HeJ mice (Tables 1 and 3). As a consequence, C3H/HeJ mice heterozygous for an exon 10 deletion in *FECH* had a significantly higher level of protoporphyrin in erythrocytes and bile than wildtype C3H/HeJ mice (Table 2), with an insignificant increase in heterozygous CBA/J mice compared to wild-type CBA/J mice. Thus, the level of expression of the nonmutant *FECH* allele is a determinant of biochemical phenotype in this mouse model of EPP.

However, the biochemical abnormality in C3H/HeJ heterozygous mice is mild compared to that in humans with clinically manifest EPP, where erythrocyte protoporphyrin levels are usually several hundred ug/dL, and in patients with severe disease several thousand ug/dL. In most humans with clinically manifest EPP, the level of expression of the nonmutant *FECH* allele is lowered by a polymorphism in intron 3 (IVS3–48c) that causes increased formation of aberrantly spliced FECH mRNA due to activation of a cryptic acceptor splice site. This causes incorporation of 63 bases of intron 3 into *FECH* mRNA, and the aberrantly spliced transcript contains a stop codon that causes it to be rapidly degraded by nonsense mediated decay. Thus the polymorphism exerts a more pronounced effect on the expression of the nonmutant *FECH* allele than occurs due to baseline expression in the mouse model. Nevertheless, among patients who carry the same mutation and the polymorphism there can be considerable variation in the severity of the phenotype, thus indicating that there are probably additional factors outside the *FECH* locus that impact phenotype in EPP, in particular transcription factors and modifier genes that might alter the level of expression of *FECH*. There are several other bone marrow genes in CBA/J mice that are differently regulated than those in C3H/HeJ mice (Tables 4 and 5). Which, if any, of these might be causing a difference in *FECH* expression cannot be determined at this time, however.

Several other investigators reported that the phenotype of a single mutation in mice was modulated by the genetic background of the strain, which was attributed to modifier genes (Montagutelli, 2000). This includes the ethylnitro-sourea-induced point mutation in *FECH* that was originally described as causing recessive inheritance of *FECH* activity in the house mouse (*FECH*^ml/Pas^/*FECH* ^ml/Pas^ mouse) (Boulechfar et al. 1993). Abitbol et al. examined three congenic strains into which the point mutation had been introduced (Abitol et al. 2005). Compared with the BALB/cByJCrl genetic background in which recessive transmission of the mutation caused severe skin lesions, anemia, jaundice and hepatic dysfunction with massive protoporphyrin deposits, C57BL/6JCrl mice developed anemia and intense liver accumulation of protoporphyrin with hepatocyte damage, but bile excretory function was not affected, and the serum bilirubin remained low. In SJL/JOrlCrl homozygous mice there was a very high protoporphyrin concentration in erythrocytes, but anemia was mild, and there were few hepatic deposits. Navarro et al. used these same three congenic strains to show a difference in the level of mitochondrial respiratory chain enzyme activities and suggested that an increase of these activities provided protection against liver disease in the EPP mice (Navarro et al. 2005). Thus, the study of different congenic mice strains with EPP may provide a means by which to identify modifier genes of phenotype in this disorder, which could help in understanding the reason for different phenotypes in human EPP.

## Figures and Tables

**Table 1:** *FECH* mRNA in different mice strains.

Strain	*FECH* mRNA ÷ 18S RNA (%)	

	bone marrow	liver
C3H/HEJ	36.9 ± 11.6	53.7 ± 5.8
129/SvEvJ	44.4 ± 5.1	62.3 ± 6.6
DBA/2J	54.4 ± 3.8	37.3 ± 4.4
BAIB/cBy	67.5 ± 8.1	50.3 ± 15.8
CBA/J	80.0 ± 16.2	126.8 ± 13.2

Mean ± SEM for 6 mice in each group. *FECH* DNA sequence in C3H/HeJ and CBA/J mice.

1 same sequence for *FECH* cDNA.

2 same sequence for proximal 1000 bases of 5′—untranslated region of *FECH* gene.

3 same base at position IVS3–48 (thymine).

**Table 2:**
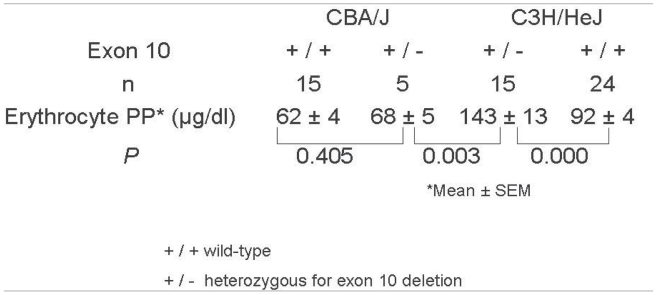
Normal FECH mRNA and FECH activity in mice.

**Table 3:** Normal *FECH* mRNA and FECH activity in mice.

	C3H/HeJ +/−	C3H/HeJ +/+	CBA/J +/+
*FECH* mRNA*			
bone marrow	138 ± 30	320 ± 59	634 ± 38
relative value	1.0	2.3	4.6
liver	430 ± 60	1078 ± 67	1798 ± 149
relative value	1.0	2.5	4.2
FECH activity**			
liver	4.4 ± 0.4	9.4 ± 0.2	10.8 ± 0.2
n	5	6	6

* copies/μg total RNA, mean ± SEM.

** pmol Zn-deuteroporphyrin per second per mg protein, mean ± SEM.

**Table 4:** Bone marrow genes upregulated in CBA/J mice compared to C3H/HeJ mice (mean fold change ≥1.5, P < 0.05).

Gene title	Fold	Gene title	Fold	Gene title	Fold
immunoglobulin heavy chain 1a (serum IgG2a)	26.5	RIKEN cDNA4930519L02 gene	2.3	caspase 3	1.9
Immunoglobulin heavy chain (gamma polypeptide)	8.5	SMC (structural maintenance of chromosomes1)-like 1 (S. cerevisiae)	2.3	platelet-activating factor acetylhydrolase, isoform1b, alpha2 subunit	1.9
Immunoglobulin heavy chain (gamma polypeptide)	7.6	jumonji, AT rich interactive domain 1D(Rbp2 like)	2.3	ADP-ribosylation factor guanine nucleotide- exchange factor 1	1.9
Son cell proliferation protein (Son), transcript variant 2, mRNA	6.2	thyroid hormone receptor interactor 12	2.3	src homology 2 domain- containing transforming protein C1	1.9
Phosphofurin acidic cluster sorting protein (Pacs1), mRNA 1	3.5	RIKEN cDNA9430041J06 gene	2.3	carnitine palmitoyltransferase 1a, liver	1.9
immunoglobulin kappa chain variable 28 (V28)///similar to immunoglobulin light chain variable region	2.9	RIKEN cDNA1810060J02 gene	2.3	RIKEN cDNA2610005L07 gene	1.9
expressed sequence AI314760	2.8	Zinc finger, SWIM domain containing 6, mRNA (cDNA clone MGC:29327 IMAGE:5025391)	2.2	sorcin	1.9
glia maturation factor, beta	2.6	high density lipoprotein(HDL) binding protein	2.1	choline kinase alpha	1.9
RNA binding motif protein 5	2.6	RIKEN cDNA9030612M13 gene	2.0	zinc finger, SWIM domain containing 6	1.8
EPM2A (laforin) interacting protein 1	2.5	RIKEN cDNA2610005L07 gene	2.0	RIKEN cDNA4932438A13 gene	1.8
expressed sequence AI585793	2.5	thyroid hormone receptor interactor 12	2.0	RIKEN cDNA2010106G01 gene	1.8
adaptor-related protein complex 3, mu 1 subunit	2.4	RIKEN cDNA2610005L07 gene	2.0	v-crk sarcoma virus CT10 oncogene homolog (avian)-like	1.8
T-cell leukemia translocation altered gene, mRNA (cDNA clone MGC:25540 IMAGE:3672301)	2.4	vacuolar protein sorting 35	2.0	hook homolog 3(Drosophila)	1.8
expressed sequence AA407175	2.4	male sterility domain containing 2	2.0	acidic (leucine-rich) nuclear phosphoprotein32 family, member A	1.8
thyroid hormone receptor associated protein 3	2.4	alpha thalassemia/mental retardation syndrome X-linked homolog (human)	2.0	chitobiase, di-N-acetyl-	1.8
GPI-anchored membrane protein 1	2.4	protein kinase, cAMP dependent, catalytic, beta	2.0	cDNA sequence BC021438	1.8
expressed sequence AW011752	2.4	pinin	2.0	tripeptidyl peptidase II	1.8
RIKEN cDNA2610018G03 gene	2.3	Toll-like receptor 4, mRNA (cDNA clone MGC:35879 IMAGE:3493732)	1.9	lipoma HMGIC fusion partner-like 2	1.8
expressed sequence AI316828	2.3	PREDICTED: histocompatibility 60 [Mus musculus], mRNA sequence	1.9	syntaxin 18	1.7
glia maturation factor, beta	2.3	RIKEN cDNA4933439C20gene	1.9	procollagen, type IV, alpha 3 (Goodpasture antigen) binding protein	1.7
RIKEN cDNA6230424C14 gene	1.7	PQ loop repeat containing	1.6		
establishment of cohesion 1 homolog 1 (S. cerevisiae)	1.7	CWF19-like 2, cell cycle control (S. pombe)	1.6		
tetratricopeptide repeat domain 14	1.7	potassium channel tetramerisation domain containing 12b	1.6		
Atpase, class VI, type11C	1.7	solute carrier family 26, member 8	1.6		
Baculoviral IAP repeat- containing 4 (Birc4), mRNA	1.7	Protein kinase C, alpha(Prkca), mRNA	1.6		
RIKEN cDNA2810474O19 gene	1.7	fusion, derived from t(12;16) malignant liposarcoma (human)	1.6		
Ndrp mRNA for neuronal differentiation related protein	1.7	zinc finger protein 146	1.6		
WD repeat domain 48	1.7	retinol binding protein4, plasma	1.6		
RIKEN cDNA4931406H21 gene	1.7	RIKEN cDNA3300001M20 gene	1.6		
RIKEN cDNA1110059P08 gene(1110059P08Rik), mRNA	1.7	ubiquitin specific peptidase 3	1.6		
ectonucleotide pyrophosphatase/phosphodiesterase 5	1.7	expressed sequence AW011752	1.6		
Period homolog 3 (Drosophila) (Per3), mRNA	1.7	influenza virus NS1A binding protein	1.6		
SEC24 related gene family, member B (S. cerevisiae) (Sec24b), mRNA	1.7	transient receptor potential cation channel, subfamily M, member 7	1.6		
nuclear receptor subfamily 2, group C, member 2	1.6	v-crk sarcoma virus CT10 oncogene homolog (avian)	1.6		
ubiquitin specific peptidase 34	1.6	poly (A) polymerase alpha	1.5		
5′-nucleotidase, cytosolic II-like 1	1.6	AF4/FMR2 family, member 1	1.5		
CCR4-NOT transcription complex, subunit 7	1.6	inhibitor of kappa light polypeptide enhancer in B-cells, kinase complex-associated protein	1.5		
ROD1 regulator of differentiation 1(S. pombe)	1.6	PREDICTED: Braf transforming gene [Mus musculus], mRNA sequence	1.5		
tripartite motif protein 30	1.6	phosphatase and tensin homolog	1.5		
MARVEL (membrane- associating) domain containing 1	1.6				
solute carrier family 2(facilitated glucose transporter), member 9	1.6				
RIKEN cDNA0610010K06 gene	1.6				
Wiskott-Aldrich syndrome protein interacting protein (Waspip), mRNA	1.6				

**Table 5:** Bone marrow genes upregulated in C3H/HeJ mice compared to CBA/J mice (mean fold change ≥1.5, P < 0.05).

Gene title	Fold	Gene title	Fold
complement receptor 2	2.3	protein kinase C, epsilon	1.6
chemokine (C–C motif) receptor 6	2.3	B-cell leukemia/lymphoma 2	1.6
Fas apoptotic inhibitory molecule 3	2.2	RIKEN cDNA 9230115F04 gene	1.6
GTPase, IMAP family member 7	2.1	phenylalanine-tRNA synthetase-like, beta subunit	1.6
Phosphatidic acid phosphatase type2B (Ppap2b), mRNA	2.0	pleckstrin homology domain-containing, family A (phosphoinositide binding specific) member 2	1.6
SH3-binding kinase 1	2.0	EMI domain containing 1	1.6
zinc finger protein 318	2.0	N-acetylglutamate synthase	1.6
phosphorylase kinase alpha 1	2.0	histocompatibility 2, O region alpha locus	1.6
chemokine (C–C motif) receptor 7	1.9	RIKEN cDNA 6430596G11 gene	1.6
CD22 antigen	1.9	caspase recruitment domain family, member 11	1.6
A kinase (PRKA) anchor protein 2	1.9	POU domain, class 2, transcription factor 2	1.6
AF4/FMR2 family, member 3 (Aff3), mRNA	1.8	Forkhead box P1 (Foxp1), mRNA	1.6
hypothetical protein A630043P06	1.8	ankyrin repeat domain 10	1.6
T-cell receptor alpha chain///RIKEN cDNA A430107P09 gene	1.8	expressed sequence AW742319	1.5
RIKEN cDNA 2310051N18 gene	1.8	gene model 1752, (NCBI)	1.5
radical S-adenosyl methionine domain containing 1	1.8	hemoglobin Y, beta-like embryonic chain	1.5
RIKEN cDNA 4921511H13 gene	1.7	SEC8 (S. cerevisiae), mRNA (cDNA clone MGC:36178 IMAGE:5355276)	1.5
PREDICTED: similar to cyclin B1 interacting protein 1 isoform a [Mus musculus], mRNA sequence	1.7	brain protein 16	1.5
TRNA splicing endonuclease 2 homolog (SEN2, S. cerevisiae) (Tsen2), mRNA	1.7	RIKEN cDNA 2700094F01 gene, mRNA (cDNA clone MGC:73433 IMAGE:6400080)	1.5
zinc finger and BTB domain containing 4	1.7	RIKEN cDNA 1810013B01 gene	1.5
Myocyte enhancer factor 2C, mRNA (cDNA clone MGC:46981 IMAGE:4500786)	1.7	Pyruvate dehydrogenase kinase, isoenzyme 1, mRNA (cDNA clone MGC:28719 IMAGE:4458562)	1.5
Fibromodulin	1.7	DEAD (Asp-Glu-Ala-Asp) box polypeptide 54	1.5
sestrin 3	1.7	lymphocyte protein tyrosine kinase	1.5
cDNA sequence BC066028	1.6	methylenetetrahydrofolate dehydrogenase (NAD+ dependent), methenyltetrahydrofolate cyclohydrolase	1.5
